# DNA barcoding-based sexual association of *Sovia lucasii* and *S*. *lii* (Lepidoptera: Hesperiidae), with description of a new subspecies

**DOI:** 10.1371/journal.pone.0183847

**Published:** 2017-08-25

**Authors:** Qing Zhai, Guo-xi Xue, Meng Li

**Affiliations:** 1 College of Plant Protection, Henan Agricultural University, Zhengzhou, Henan, the People’s Republic of China; 2 School of Food and Bioengineering, Zhengzhou University of Light Industry, Zhengzhou, Henan, the People’s Republic of China; Tierarztliche Hochschule Hannover, GERMANY

## Abstract

Both sexes of two sympatric and sexually dimorphic species, *Sovia lucasii* (Mabille, 1876) and *S*. *lii* Xue, 2015, are associated based on DNA barcoding using the COI (mitochondrial cytochrome coxidase I) gene. The females are thus identified for the first time, and their wing patterns and genitalia are described and illustrated for the convenience of morphological identification hereafter. A new subspecies, *S*. *lucasii minor* ssp. nov., from the northeastern and eastern parts of the Sichuan Basin of China, is reported. External and genital differences between the new taxon and the nominate subspecies, which is distributed in western Sichuan and newly discovered in northwestern Guangxi, are illustrated and discussed.

## Introduction

Sexual dimorphism, which is common in many Lepidopteran insect groups, impedes the taxonomic study of the unidentified sexes of closely related species [1–3]. In Hesperiidae, most species were described based upon male specimens, and until now, the females of many species have not been reported yet, because the identification of female specimens is challenging, especially for the sexually dimorphic species that have similar wing patterns, and the discovery of the females of some hesperid species in the wild is rather difficult [4].

During the course of our study on the skippers of the Qinling-Daba Mountains, a series of specimens were collected, including the males of *Sovia lii* Xue, 2015 and its sympatric species, *S*. *lucasii* (Mabille, 1876), as well as two forms of females. Judging from the external morphological characters, the females should belong to *S*. *lii* and *S*. *lucasii* respectively, although their wing patterns clearly demonstrate sexual dimorphism. To correctly associate the females with their male counterparts, partial COI (mitochondrial cytochrome coxidase I) genes of these specimens are sequenced and compared, and the two forms of females are successfully associated with their conspecific males in the phylogenic tree. Thereafter, wing patterns and genitalia of the identified *S*. *lii* and *S*. *lucasii* females are described and illustrated, making possible the morphology-based identification of the two species’ female specimens.

Xue et al. [5] noted that the population of *Sovia lucasii* in the Daba Mountains exhibits a smaller body size than that of western Sichuan and may represent a separate subspecific race. In this study, external and genital characters of both sexes of *S*. *lucasii* from these two areas are compared. The results show that the two groups have steady morphological differences. Therefore, the population with a smaller body size is described as a new subspecies in this paper.

## Materials and methods

### Ethics statement

The permission for fieldwork in Cenwanglaoshan, Tianlin County, Guangxi Zhuang Autonomous Region was issued by the Administration Bureau of Cenwanglaoshan National Nature Reserve. No specific permissions were required for other locations because they are not in protected areas or on private land. Field studies in this work did not involve endangered or protected species.

### DNA extraction and sequence analysis

Genomic DNA was extracted from 1–2 legs (dry or in 100% ethanol) of each specimen (Table 1) using an animal tissue DNA extraction kit (Huier nanotechnology, Luoyang, China) according to the manufacturer’s protocol. The DNA was dissolved in a 100 μl elution buffer and stored at -20°C. Partial COI genes were amplified by PCR with the universal primers Ron (5’-GGATCACCTGATATAGCATTCCC-3’) and Nancy (5’-CCCGGTAAAATTAAAATATAAACTTC-3’) [6]. For each PCR, a 20 μl mixture containing 2 μl template DNA, 2 μl 10×PCR buffer (Sangon biotech, Shanghai, China), 0.3 U Taq polymerase (Sangon biotech, Shanghai, China), 2.5 mM MgCl_2_, 0.5 μl dNTPs (10 mM each) and 0.5 μl (10 μM) forward and reverse primers was used under the reaction conditions described by Hebert et al. [7] on a thermal cycler (AB, the USA). The ~500bp PCR products were sequenced by Sangon biotech, Shanghai, China. DNA sequences were first aligned in Clustal X 2.0 software [8], and then a neighbor-joining (NJ) tree based on the Kimura-2-parameter distance model [9] was generated by MEGA6 software [10] to quantify sequence divergences among specimens. Nodal support of the NJ tree was estimated by 1000 bootstraps. All sequences obtained in this paper were deposited in GenBank (Table 1).

**Table 1:** List of specimens used for sequencing.

Species	Locality	Coordinates	Specimen ID	Sex	Date	Accession number
Unidentified female, form 1	Zhanghe, Lan’gao County, Shaanxi Province	32°05′N 108°54′E	female_form1_Shaan1	female	21.VII.2013	KY776498
	Qinghe, Kangxian County, Gansu Province	33°12′N 105°51′E	female_form1_GS1	female	18.VII.2014	KY776498
Unidentified female, form 2	Zhanghe, Lan’gao County, Shaanxi Province	32°03′N 108°53′E	female_form2_Shaan1	female	29.VII.2012	KY776499
	Zhanghe, Lan’gao County, Shaanxi Province	32°03′N 108°53′E	female_form2_Shaan2	female	30.VII.2012	KY776501
	Zhanghe, Lan’gao County, Shaanxi Province	32°03′N 108°53′E	female_form2_Shaan3	female	29.VII.2012	KY776501
	Emei Mountain, Sichuan Province	29°33′ N 103°21′ E	female_form2_Sich1	female	6.VIII.2014	KY776500
*Sovia lii* Xue, 2015	Zhanghe, Lan’gao County, Shaanxi Province	32°05′N 108°54′E	lii_Shaan_M1	male	21.VII.2013	KY776498
	Zhanghe, Lan’gao County, Shaanxi Province	32°05′N 108°54′E	lii_Shaan_M2	male	21.VII.2013	KY776498
	Shangzhu, Zhenping County, Shaanxi Province	31°56′N 109°25′E	lii_Shaan_M3	male	25.VII.2014	KY776498
	Qinghe, Kangxian County, Gansu Province	33°12′N 105°51′E	lii_GS_M1	male	18.VII.2014	KY776498
*Sovia lucasii* (Mabille, 1876)	Zhanghe, Lan’gao County, Shaanxi Province	32°05′N 108°54′E	luca_Shaan_M1	male	21.VII.2013	KY776499
	Emei Mountain, Sichuan Province	29°33′ N 103°21′ E	luca_Sich_M1	male	7.VIII.2014	KY776500
	Emei Mountain, Sichuan Province	29°33′ N 103°21′ E	luca_Sich_M2	male	6.VIII.2014	KY776500
	Gongyihai, Shimian County, Sichuan Province	29°02′ N 102°22′ E	luca_Sich_M3	male	10.VII.2015	KY776501
	Gongyihai, Shimian County, Sichuan Province	29°02′ N 102°22′ E	luca_Sich_M4	male	10.VII.2015	KY776501
	Heizhugou, Ebian County, Sichuan Province	29°02′ N 103°0′ E	luca_Sich_M5	male	8.VII.2015	KY776501
	Cenwanglaoshan, Tianlin County, Guangxi Zhuang Autonomous Region	24°29′ N 106°17′ E	luca_GX_M1	male	9.VI.2015	KY776501
	Cenwanglaoshan, Tianlin County, Guangxi Zhuang Autonomous Region	24°25′ N 106°22′ E	luca_GX_M2	male	9.VI.2015	KY776501
*Sovia subflava* (Leech, 1894)	Balagezong, Zhongdian County, Yunnan Province	28°19′ N 99°30′ E	subflava_YN	male	23.VI.2014	KY709702

### Morphological study

In addition, the following specimens of *Sovia lucasii* were also examined and compared with the new subspecies: 2♂♂, Hanyuan, Sichuan, 1200–1300 m, VI.2009, Zhi-bing Chen leg.; 4♂♂, Jiulinggang, Emei Mountain, Sichuan, 6–7.VIII.2014, Zi-hao Liu leg.; 1♂, Xiling Snow Mountain, Dayi, Sichuan, 21.VII.2014, Si-yao Huang leg.; 1♂, Heizhugou, Ebian County, Sichuan, 7.VII.2015, Chun-hao Wang leg. Materials of the new subspecies were listed in the type series. All the specimens used in this study were deposited in the School of Food and Bioengineering at the Zhengzhou University of Light Industry in Zhengzhou, China, and in the private collections of Mr. Yu-fei Li (Xi’an) and Mr. Jian-qing Zhu (Shanghai).

Images of adults were taken with a Canon PowerShot G16 digital camera. Genitalia were examined and photographed using an Olympus SZX7 stereomicroscope after clearing in a 10% NaOH solution. Image post-processing was accomplished with Adobe Photoshop CS 8.0.1. The Comstock-Needham venation system was used in this paper. The terminology of male genitalia mainly follows Shirôzu [11].

### Nomenclatural acts

The electronic edition of this article conforms to the requirements of the amended International Code of Zoological Nomenclature, and hence the new names contained herein are available under that Code from the electronic edition of this article. This published work and the nomenclatural acts it contains have been registered in ZooBank, the online registration system for the ICZN. The ZooBank LSIDs (Life Science Identifiers) can be resolved and the associated information viewed through any standard web browser by appending the LSID to the prefix “http://zoobank.org/”. The LSID for this publication is: urn:lsid:zoobank.org:pub:55D3B445-85B7-4A27-BE0F-96E0218A0489. The electronic edition of this work was published in a journal with an ISSN, and has been archived and is available from the following digital repositories: PubMed Central, LOCKSS.

## Results

### Sexual association of *Sovia lucasii* and *S*. *lii* via DNA

**Fig 1:**
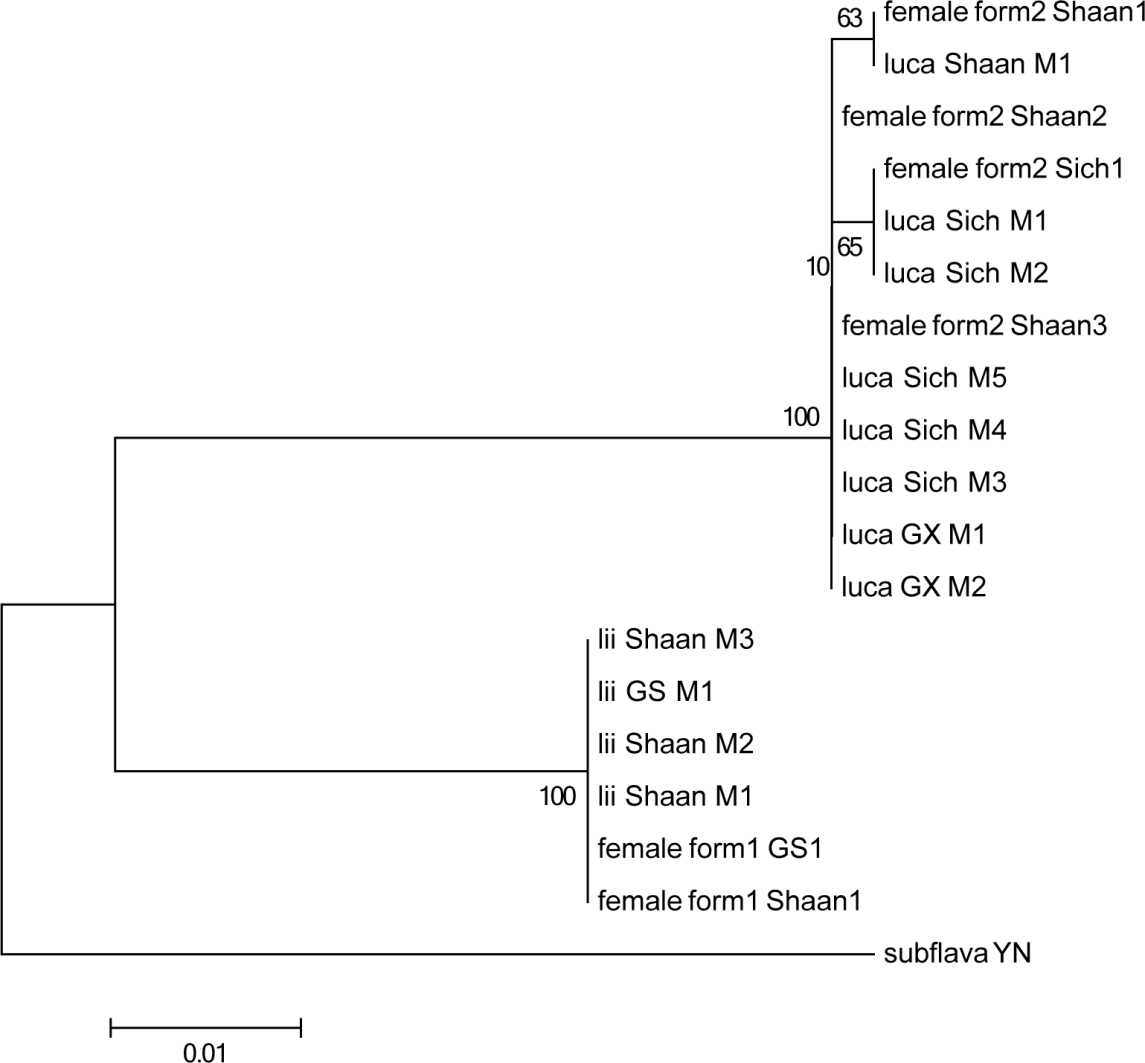
NJ tree for the partial mitochondrial COI sequences from 19 individuals of *Sovia*.

All specimens successfully generated a 455 bp partial COI sequence via PCR and sequencing. The sequence divergences are shown in Fig 1. In the NJ tree, the two forms of female specimens were respectively assigned to the clades of the male individuals of *Sovia lucasii* and *S*. *lii* with a sequence identity of 99% or 100% (Fig 1). Therefore, the female form 1 was recognized as *S*. *lii*, and the female form 2 was recognized as *S*. *lucasii*.

### Description of *Sovia lucasii minor* Xue, ssp. nov.

urn:lsid:zoobank.org:act:25543A5B-32A3-42FC-AF12-9EFDBC66E92C

Holotype: 1♂, Zhanghe, Lan’gao, Shaanxi, 1100 m, 21.VII.2013, Guo-xi Xue, Wen-hao Nan & Xing-long Jia leg.

Paratypes: 1♂, same data as holotype; 1♂5♀♀, same locality as holotype, 1400–1600 m, 29–30.VII.2012, Yu-fei Li leg.; 1♀, Qingshui, Zhenba, Shaanxi, 1500–1700 m, 6.VIII.2014, Yu-fei Li & Zhao-dong Li leg.; 4 ♂♂, Houhe, Wufeng, Hubei, 1200m, 8–9.VII.2013, Jian-qing Zhu leg. (examined by photo).

**Fig 2:**
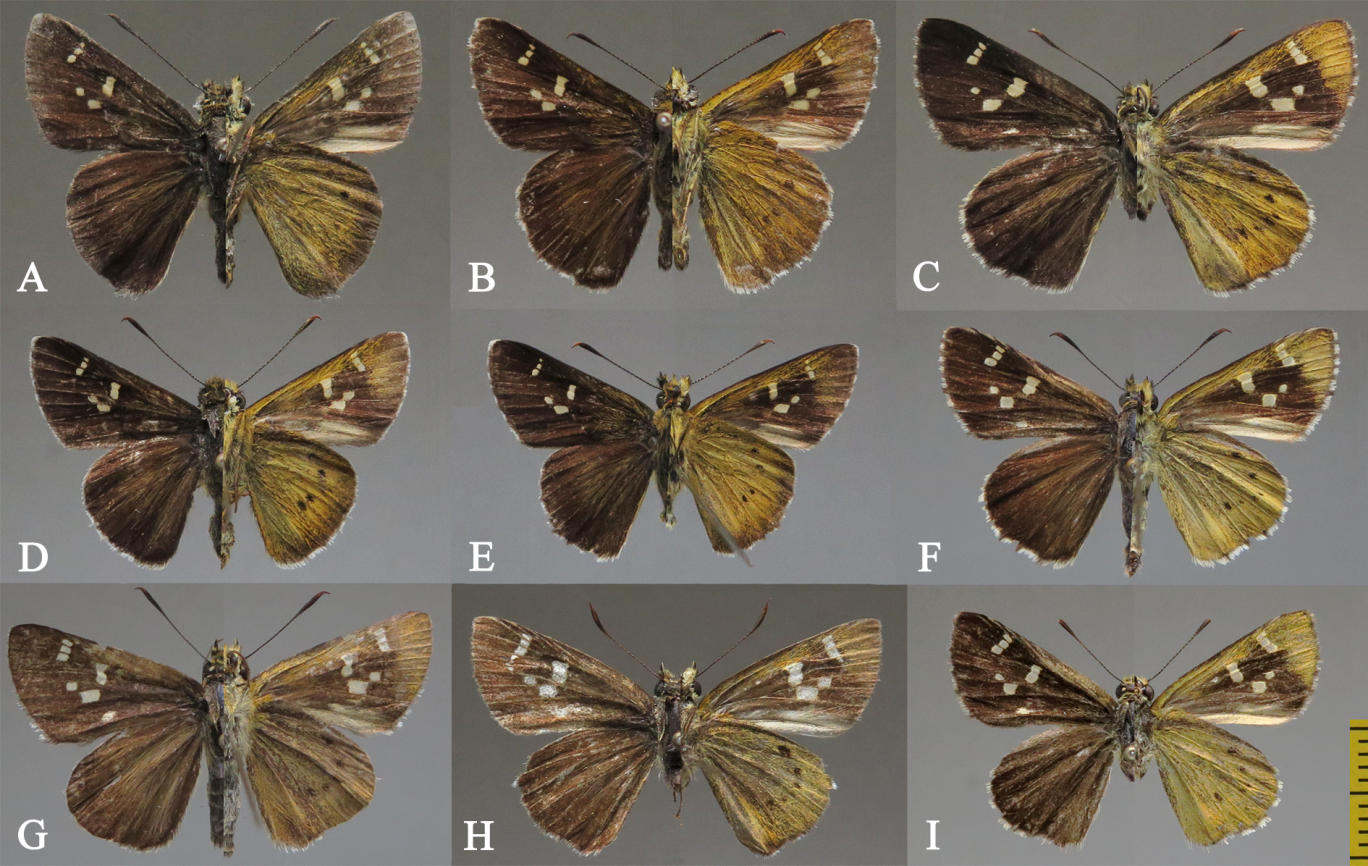
Adults of *Sovia*. (A–C) *S*. *lucasii lucasii*; (D–F, I) *S*. *lucasii minor*; (D) Holotype; (E, F, I) Paratypes; (G–H) *S*. *lii*. (A, B, D, E) Male; (C, F–I) Female. Scale bar = 1 cm.

**Fig 3:**
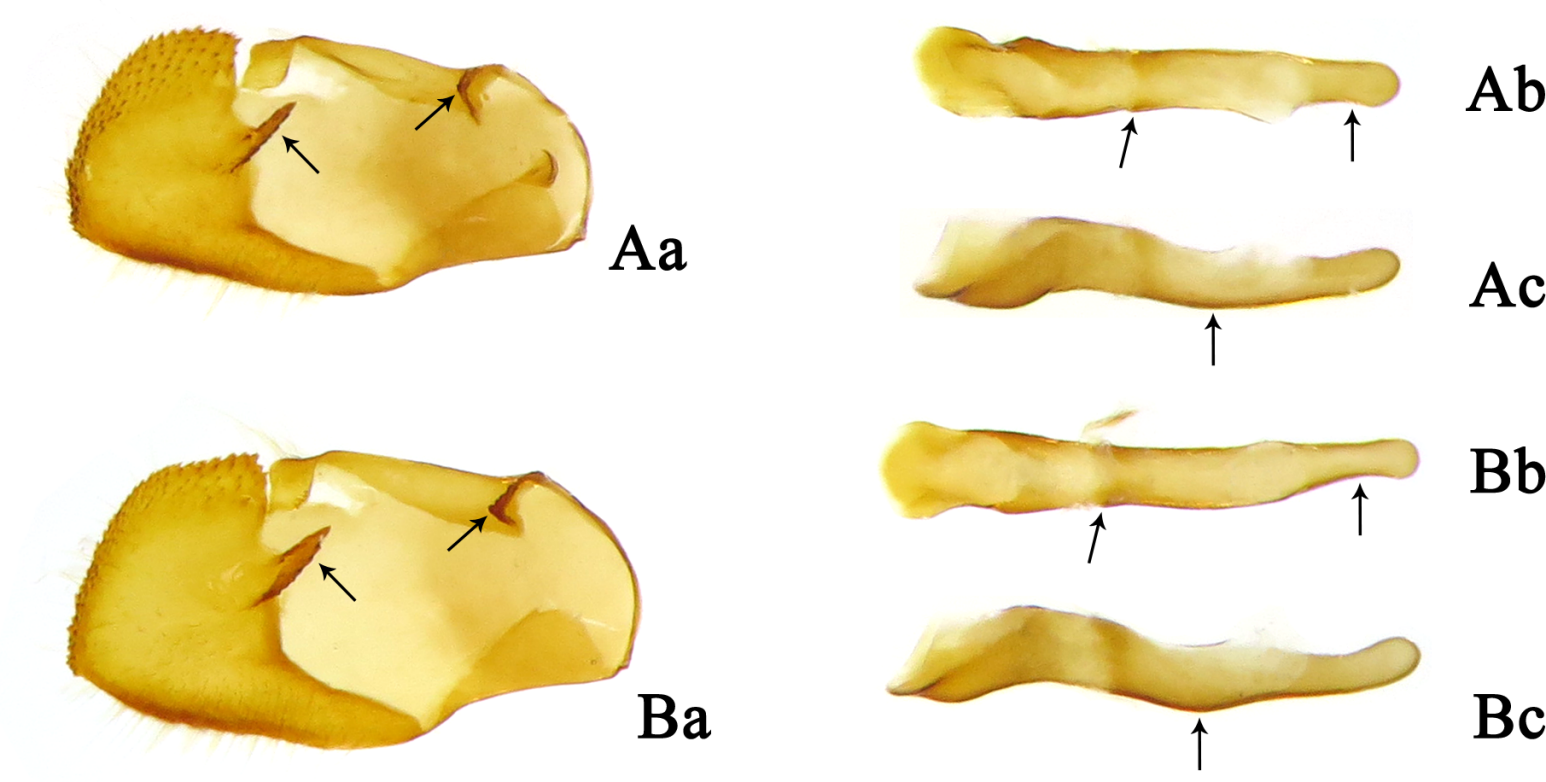
Male genitalia of *Sovia*. (A) *S*. *lucasii minor*; (B) *S*. *lucasii lucasii*. (a) left valva, inner side; (b) aedeagus, dorsal view; (c) aedeagus, lateral view.

**Fig 4:**
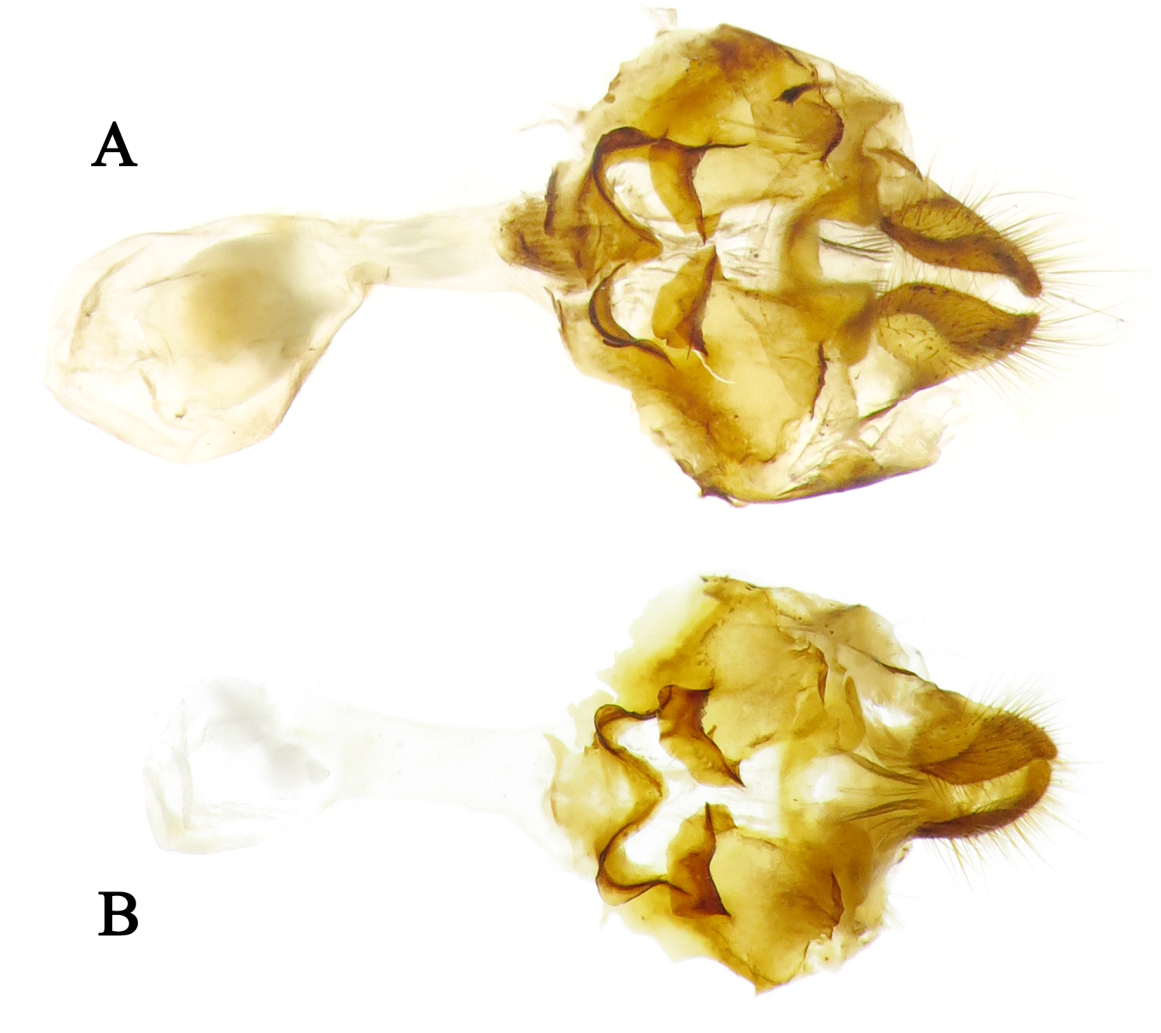
Female genitalia of *Sovia*, ventral view. (A) *S*. *lucasii lucasii*; (B) *S*. *lucasii minor*.

Diagnosis. Externally, the new subspecies (Fig 2D–2F and 2I) is almost the same as *Sovia lucasii lucasii* (Fig 2A–2C), but it is distinguishable from the latter by a smaller size (forewing length: 14–15 mm (n = 7) vs. 15–17 mm (n = 15) in males, 15–16 mm (n = 6) vs. 17.5 mm (n = 1) in females). The male genitalia of the new subspecies are very similar to those of the nominate subspecies (Figs 23–26 in [5]), except for the following differences (Fig 3): the footstalk is blunt (pointed in *S*. *lucasii lucasii*); the harpe is thinner (robust in *S*. *lucasii lucasii*); the middle of the ventral margin of the aedeagus is slightly curved (conspicuously convex in *S*. *lucasii lucasii*); coecum and subzonal sheath of the aedeagus are shorter than those of *S*. *lucasii lucasii*. Compared with *S*. *lucasii minor*, the female of the nominate subspecies (Fig 2C) has the forewing spot in space cu_1_ rectangular, nearly equidistant from the one in space m_3_ and the cell spot (closer to the spot in space m_3_ in the new subspecies); in its genitalia (Fig 4A), the gap on the posterior edge of the lamella postvaginalis is quadrate (U-shaped in the new subspecies), the middle plate of the lamella antevaginalis is more pointed, and the two gaps of the W-shaped posterior edge are much wider.

Since the differences between the male of the new subspecies and that of *S*. *lucasii lucasii* have been discussed above, it is unnecessary to give a routine description. Only the female of the new subspecies is described as follows.

Female (Fig 2F and 2I). Antennae: approximately 8.5 mm in length, dorsal side dark brown, ventral side checkered; apiculus brownish-red, hocked and sharply pointed. Labial palpi: second segment covered with yellowish hairs and scales; third segment porrect, thicker than shaft of antennae, black dorsally, yellowish ventrally, with a blunt point. Thorax and abdomen: dorsal side black, ventral side covered with yellowish hairs. Forewing: 15–16 mm in length, dorsal side dark brown, spots white, semi-hyaline; apical spots in spaces r_3_–r_5_ arranged in a line; cell spot elongated, placed near discocellular, perpendicular to costa; spot in space cu_1_ quadrate, closer to the small spot in space m_3_ than to cell spot; below the gap between spots in space cu_1_ and cell, there is a small oblong spot in the lower half of space cu_2_ located near vein 2A; ventral side of forewing with costal and apical area bright yellow, discal area dark brown, and dorsumal area gray, spots as in dorsal side. Hindwing: dorsally dark brown and unmarked, dorsumal area covered with hairs; ventral side broadly clad with bright yellow scales, a series of submarginal spots from space sc+r_1_ to cu_1_ parallel to termen. Cilia checkered.

Female genitalia (Fig 4B). Papillae anales almost triangular, covered with long hairs. Distal half of lamella postvaginalis sclerotized, middle of its posterior edge with a U-shaped gap. Central part of anterior half of lamella postvaginal is membranous, with a sclerotized plat on each side with the anterior edge thickened and irregularly arcuate. Distal edge of lamella antevaginal is W-shaped, middle plate triangular with a round top. Ductus bursae tube-like; bursa copulatrix bursiform, without signum.

Etymology. The scientific name is referring to the smaller body size of the new subspecies.

**Fig 5:**
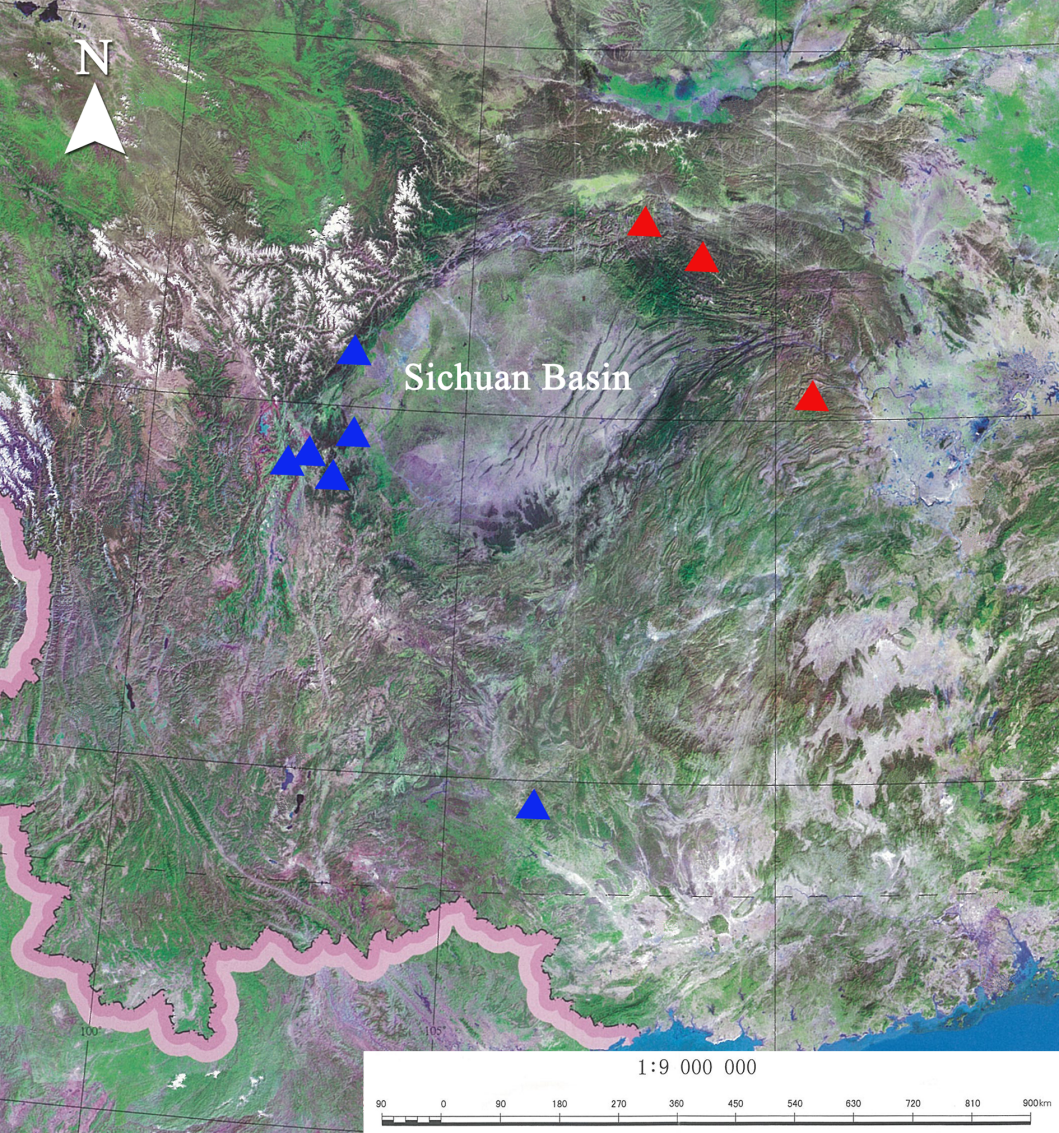
Distribution map of *Sovia lucasii minor* (red triangle) and *S*. *lucasii lucasii* (blue triangle) based upon a map scanned from [12].

Distribution (Fig 5). Southern Shaanxi, Northwestern Hubei.

Remarks. The discovery of *Sovia lucasii* in northwestern Guangxi reveals a more extensive distributional range of the taxon. Although there is a conspicuous distance between northwestern Guangxi and western Sichuan, the morphological characters of the examined materials indicate that the population from northwestern Guangxi belongs to the nominate subspecies rather than *S*. *lucasii minor*.

### Description of the female of *Sovia lii* Xue, 2015

Adult (Fig 2G–2H). Antennae approximately 9.5 mm in length, forewing 15–16 mm in length. Wing pattern similar to that of female *Sovia lucasii*, but forewing spots in spaces m_1_, cu_1_ and cell much closer, cell spot almost perpendicular to dorsum.

**Fig 6:**
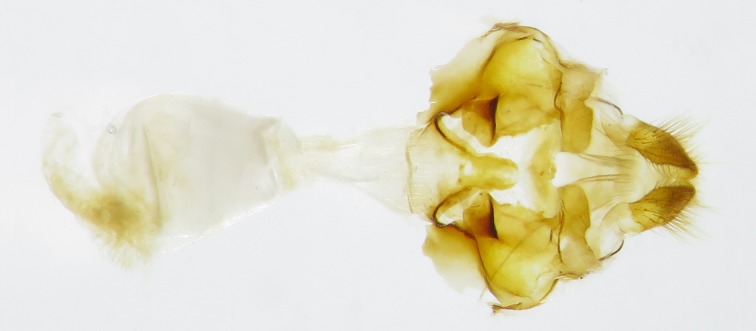
Female genitalia of *Sovia lii*, ventral view.

Female genitalia (Fig 6). Papillae anales almost triangular, covered with long hairs. Distal half of lamella postvaginalis sclerotized, medial gap of posterior edge U-shaped but the bottom wider. Central part of anterior half of lamella postvaginalis membranous, sclerotized plat on each side with anterior area thickened and protruding inward. Basal half of lamella antevaginalis tapered, distal half tongue-like. Ductus bursae tube-like; bursa copulatrix bursiform, without signum.

## Discussion

The identification of female specimens is rather difficult for some sexual dimorphic groups in Lepidoptera, because most of the species are described from male specimens. Additionally, similar species are often found to be sympatric [5, 13], and thus the keys usually based upon male characteristics are inapplicable for females. Similarly, for sexual dimorphic species identified using female type materials, the identification of the corresponding males becomes a problem as well [2]. In some cases, the opposite sexes of one dimorphic species have been described as two different species [1–3], causing taxonomic confusion. DNA barcoding, which has been widely used to distinguish cryptic species [6, 14] and to investigate biodiversity [15], was reported as useful for revealing sexual dimorphism in Tortricidae [16] and Hesperiidae [17]. In this study, it is also found to be quite helpful to distinguish females of similar sexual dimorphic species in Hesperiidae. It can be expected that the sexual association of dimorphic lepidopteran species and other insects, important work for research in taxonomy, phylogeny and biology, will be greatly accelerated by DNA barcoding in the future.
